# Effect of different angulations and collar lengths of conical hybrid implant abutment on screw loosening after dynamic cyclic loading

**DOI:** 10.1186/s40729-018-0149-z

**Published:** 2018-12-03

**Authors:** Mai Ahmed Yousry El-Sheikh, Tamer Mohamed Nasr Mostafa, Mohamed Maamoun El-Sheikh

**Affiliations:** Prosthodontic Department, Faculty of Dentistry, Tanta University, Elgeish St., Tanta, Egypt

**Keywords:** Removal torque loss, Abutment angulation, Abutment collar height, Dynamic cyclic loading, Digital torque gauge, Screw loosening

## Abstract

**Background:**

The purpose of this in vitro study was to evaluate the effect of different angulations and collar lengths of the implant abutment on screw loosening by measuring removal torque value (RTV) before and after dynamic cyclic loading using digital torque gauge.

**Methods:**

A total 90 sets of 4.5 mm diameter × 10 mm length bone level implants with conical hybrid connection were used. They were divided equally according to abutment angulation, into three groups: GI 0° abutment, GII 15° abutment, and GIII 25°. Each group was divided into two subgroups, 15 each, according to collar height: subgroup A (2 mm) and subgroup B (4 mm). Each implant and abutment assembly was positioned vertically in the center of the acrylic resin block using stainless steel cylindrical split mold. Initial analysis was made by abutment screw tightened with 30 Ncm torque twice with 10-min intervals using a digital torque gauge. RTV before and after cyclic loading of the abutment screws were measured in newton centimeter using digital torque gauge. One hundred thousand cycles of eccentric dynamic cyclic loading, at 130 N at a rate of 1 Hz, were applied 5 mm away from the central axis of the implant fixture. Percentage of removal torque loss (%RTL) before and after dynamic cyclic loading were calculated and statistically analyzed using the SPSS version 20.

**Results:**

For GI, %initial RTL was 25.0 ± 1.5% and decreased significantly after loading (23.5 ± 2.3%). For GII, %initial RTL was 25.5 ± 1.4% and increased significantly after loading (33.4 ± 3.7%). For GIII, %initial RTL was 25.944 ± 1.2% and increased significantly after loading (40.1 ± 5.1%). There was significant effect on screw loosening for abutment angulations and collar lengths.

**Conclusion:**

Within the limitations of this study, results suggested that screw loosening increases with increasing abutment angulations and collar lengths after dynamic cyclic loading.

## Introduction

Successful implant therapy requires a dynamic equilibrium between biological and mechanical factors. The mechanical factors are generally considered multifactorial which are involved in the success of implant rehabilitation. Majority of implant complications nowadays are caused by mechanical factors rather than the implant itself. Mechanical complications of the implant-prosthetic system include loosening and fracture of the maintaining screw, micromovements, fixture fracture, abutment fracture, and fracture of over structure [1].

It was reported that abutment screw loosening is the most common mechanical complication surpassed by loss of osseointegration [2]. Loosening and fracture are potential problems for implant abutments and their fastening screws [3]. Incidence of screw loosening was up to 12.7% in single crowns and 6.7% in fixed partial dentures [4].

Several complications may arise because of loose retaining or abutment screws as granulation tissue between the loose abutment and the implant, leading to fistulae formation and infection of the soft tissue. In addition, loose screws are more liable to fracture under load, leading to long-term prosthesis complications [2].

The whole concept of the dental implant mechanics has been designed in such a way as to have a weak link that will be the first component that will fail in case the system is overloaded. The screw used to fasten the abutment to the implant usually represents this weak link [3].

Considering that the union between prosthesis and implant is promoted by a screw joint, the aim to tight the abutment screw is to keep the components together [5]. A screw is tightened by applying torque as a clamping force to provide a stable joint between the abutment and implant fixture. This clamping force is also known as the preload, which elongates the screw within the material then elastically recovered, increasing the strength with which the abutment and implant are pulled together [6].

When the implant set is submitted to functional loads, occlusal forces to the connection are concentrated at the abutment screw; consequently, the optimum preload is critical for joint stability and to avoid screw loosening [7].

Several factors related to screw design and fabrication can affect the risk of abutment or prosthetic screw loosening in a metal-to-metal screw system; these primarily are related to preload which by itself is affected by multiple factors: torque magnitude, screw head design, thread design, and number and composition of metal [8]. There are some factors that can affect initial torque loss, including tightening torque value, implant system, abutment screw material, errors in casting of metallic alloys, repeated tightening/loosening cycles of the screw, and improper insertion torque. These factors can reduce the frictional fit between the screws and internal threads of the implant, which may lead to screw loosening [9, 10].

Also screw loosening may be caused by inadequate tightening torque, settling of implant components, inappropriate implant position, inadequate occlusal scheme or crown anatomy, poorly fitting frameworks, improper screw design/material, increased abutment angulations, increased collar length, and heavy occlusal forces [11].

Ideally, dental implants should be aligned vertically with the axial forces. When the long axis of the implant fixture and the long axis of the planned prosthetic tooth are not aligned, due to improper jaw relationship or compromised osseous anatomy, angled abutment is often the abutment of choice for prosthodontic restorations [4]; it helps to avoid vital anatomical structures [12]. Angled abutments are used in all-on-four and all-on-six approaches in completely edentulous patients [13]. They can be used for esthetics reasons [8]. Angled abutments reduce treatment time, fees, and the need to perform guided bone regeneration procedure [14].

Kallus et al. [15] demonstrated prototype angled abutments of the Branemark. Nowadays, angled abutments vary from 15 to 45° angulation. Researches showed that angled abutment developed transverse force under loads in the direction of angled abutment resulting in off-axis forces. When functional or parafunctional load is applied to angled abutment, it generates micromovement which might play a role in screw loosening [4].

Collar length is the distance between the implant platform and the gingival margin. Sometimes, significant vertical space that has not been corrected with vertical ridge augmentation may necessitate selection of longer abutments, which would lead to an increased vertical cantilever. Furthermore, selection of the length of abutment collar would be affected if different distances between the implant platform and the gingival margin exist. Despite consistent occlusogingivial dimension, the thickness of soft tissue around the abutment affects abutment collar length selection. Therefore, in posterior regions where reduction of surrounding soft tissues thickness does not interfere with esthetic results, this reduction may be beneficial from a biomechanical point of view [9]. Selection of the suitable abutment collar length from a prosthetic/esthetic point of view is influenced by the length of the implant collar used. Abutment collar length is determined based on the height of the emergence profile and prosthetic restoration type (cemented, screw-retained, or overdenture) [16].

Abutment selection according to collar length index is a critical mechanical factor; selection of longer abutments leads to an increased vertical cantilever which acts as a force magnifier [8]. Vertical cantilever designs increase forces on screws due to the lever effect and, therefore, should be avoided [17, 18]. Although increased restorative vertical space with longer collar length could play a role in screw loosening, there is no certain evidence that increase in abutment collar length can affect screw loosening. However, considering the abutment height from implant platform to the top of abutment (including abutment collar), an increase in the collar length might result in an increase in the vertical cantilever. To reduce the possibility of screw loosening, reducing the cantilever length has been recommended [9].

The application of dynamic cyclic loading is used to simulate masticatory function mimic oral cavity that might lead to a failure of implant–abutment connection. Also, it is a reliable method to test the effect of mechanical fatigue on the implant–abutment stability [4].

To date, there are limited publications regarding the investigation of screw loosening according to abutment angulations and collar lengths before and after dynamic cyclic loading. Sethi and colleagues [19] noted no implant fractures or screw loosening during a 96-month monitoring period for 2261 implants (*N* = 467 patients) with angulations ranging from 0 to 45°. While Asvanund and colleagues [4] addressed screw loosening with increasing abutment angulations, Siadat and coworkers [9] concluded that increase in height of the abutment collar could adversely affect the torque loss of the abutment screw.

So, the aim of this in vitro study was to evaluate the effect of different angulations and collar lengths of the implant abutment on screw loosening by measuring removal torque value before and after dynamic cyclic loading using digital torque gauge.

## Materials and methods

**Fig. 1:**
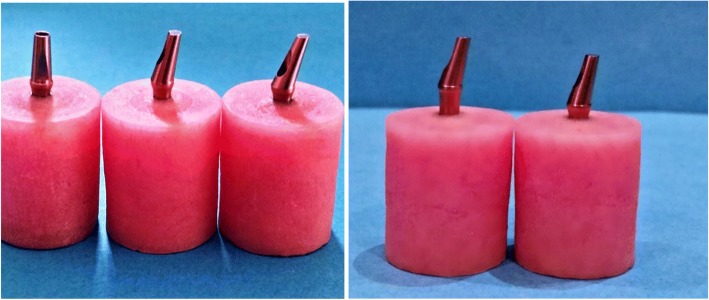
Different abutment angulations and collar lengths

Total 90 stock titanium abutments (Anyridges; MEGAGEN, Seoul, Korea) with different angulations and collar lengths were used in this study and divided into three groups, 30 each according to the degree of abutment angulations GI 0° abutment, GII 15° abutment, and GIII 25°. Each group was divided into two subgroups, 15 each according to implant abutment collar lengths: subgroup A (2 mm) and subgroup B (4 mm). For each sample conical hybrid connection implant, 4.5 mm diameter × 10 mm length bone level implants with platform-switching were used. Titanium abutment screw was used (Fig. 1).

Ninety auto-polymerized acrylic resin blocks were prepared for this study using a stainless steel split cylindrical mold with 20 mm length, 20 mm width, and 2.5 in. thickness. A stainless steel base was made wider than the split cylinder, so it can be seated inside this base. At the center of the base, small opening with the same diameter of implant abutment was made so it can help implant abutment assembly to be centralized vertically in the acrylic resin block. Split cylindrical mold was cleaned and dried; then, vaseline was applied into the whole internal surface to ensure separation of acrylic block from the mold. Implant fixture and abutment were screwed through the hole in the stainless steel base.

**Fig. 2:**
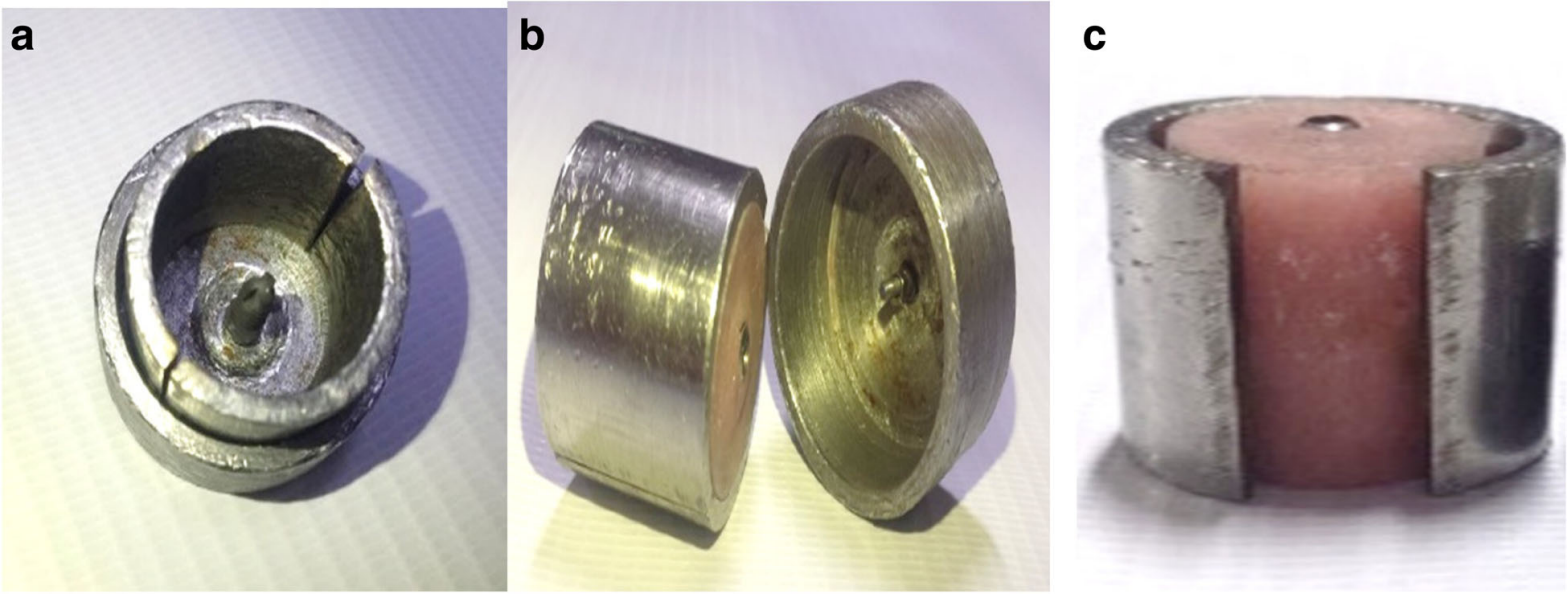
**a** Stainless steel split cylindrical mold with implant fixture screwed to abutment. **b** Implant fixture unscrewed from abutment after polymerization. **c** Implant fixture centralized vertically and perpendicular to the base with platform flushed with resin block level

The auto-polymerized acrylic resin powder and liquid were mixed according to the manufacturer’s recommendation, poured inside the split cylindrical mold, and left for polymerization. After polymerization, implant fixture was unscrewed from abutment to separate the split cylinder from the stainless steel base; then, the cylinder was removed showing the acrylic resin block with implant fixture centralized vertically and perpendicular to the base and the platform was flushed with acrylic resin block level. Finishing and polishing were made, using micromotor, by red and white stone (Fig. 2).

**Fig. 3:**
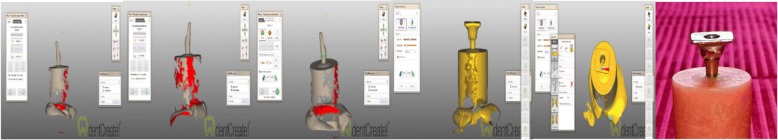
3D scanning for abutment and designing for metal tube

Ninety stock titanium abutments were selected with post length of 7 mm; each abutment had titanium screw with same length, diameter, thread, and head design for each group. For each selected abutment, metal tube was fabricated to fit accurately on the abutment. The titanium abutment was mounted on the fixture which was prepared to be scanned with a 3D scanner (Smart optics, England). The desired design and dimensions of metal tube was designed by CADCAM software (Dentcreate, Exocad) in wax (CopraDur, White peaks dental solution, Germany), with flat occlusal surface (10 mm in diameter) which was parallel to the horizontal plane and perpendicular to the implant fixture long axis to permit contact with the testing machine piston in a flat horizontal plane. In the center of the flat occlusal surface, a small rounded hole was designed exactly opposite to the abutment screw hole that facilitates screw driver accessibility for easy tightening and removal. Then, this accurately designed wax pattern was casted to a nickel chromium alloy tube (Fig. 3).

Self-adhesive resin cement (G- CEM, USA) was used for fixing of metal tube to the abutment. A customized rigid metal mounting jig was designed, as a holding device, to ensure solid fixation of the sample while recording the measures.

### Recording RTV before loading

Abutment screws were tightened in all the groups to 30 Ncm using a digital torque gauge (HTG2 - 200Nc, IMADA, Toyohashi, Japan) according to the manufacturer’s recommendations to ensure an accurate application of reproducible force to each abutment screw every time for standardization. Ten minutes after first torque application, all screws were retightened to the same tightening torque (30 Ncm). Ten minutes later, RTV before loading was measured and recorded.

### Recording RTV after loading

At each group, after measuring the initial removal torque value, the abutment screw was tightened again to the recommended torque value (30 Ncm).

The acrylic resin block was firmly mounted in a holder of the lower fixed compartment of a computer-controlled universal testing machine (Model 3345; Instron Industrial Products, Norwood, MA, USA) for 100,000 cycles of eccentric dynamic cyclic loading.

**Fig. 4:**
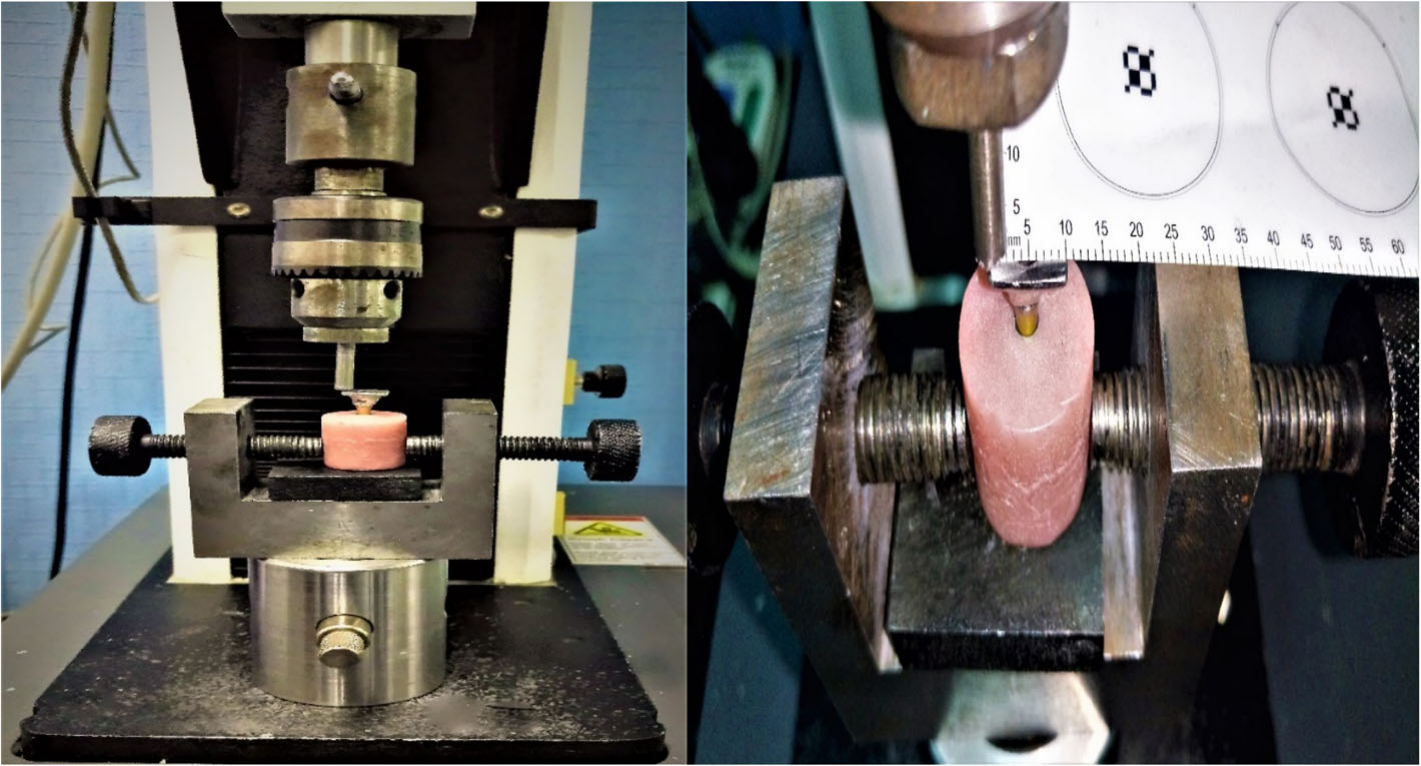
Application of cyclic loading with universal testing machine

Dynamic cyclic loading was performed with a metallic rod with a round tip which was attached to the upper movable compartment of the machine, under a load of 130 N with contact time between the rod and the metal tube 0.2 s at a rate of 1 Hz which simulates the tooth contact duration of each masticatory cycle. The load was perpendicular to the metal tube and 5 mm away from the center axis of the implant (Fig. 4). After that, the acrylic resin block with fixture and abutment was transferred again to the metal jig to measure RTV after loading.

### Calculation of RTL ratio of abutment screw before and after dynamic cyclic loading

Screw loosening of each assembly (implant–abutment) was analyzed by measuring RTV before and after dynamic cyclic load by using the digital torque gauge. RTL can be an indicator of how much loosening takes place.

Each RTL ratio was calculated using the following formula:

Removal torque loss ratio before loading (%initial RTL)

$$ =\frac{\mathrm{Tightening}\ \mathrm{torque}-\mathrm{removal}\ \mathrm{torque}\ \mathrm{before}\ \mathrm{loading}}{\mathrm{Tightening}\ \mathrm{torque}}\times 100 $$

Removal torque loss ratio after loading (%postload RTL)

$$ =\frac{\mathrm{Tightening}\ \mathrm{torque}-\mathrm{removal}\ \mathrm{torque}\ \mathrm{after}\ \mathrm{loading}}{\mathrm{Tightening}\ \mathrm{torque}}\times 100 $$

Removal torque loss ratio between before and after loading (%difference between initial and postload RTL)

Student *T* test and ANOVA were used to compare mean of %initial and %postload RTL between different groups and subgroups followed by pair-wise Tukey’s post hoc tests at 0.05 level of significance. Differences between mean greater than Tukey post hoc value were considered significant (*P* < 0.05). Statistical analysis was performed by using SPSS program version 20 (SPSS 20; Inc. Chicago, USA).

## Results

**Fig. 5:**
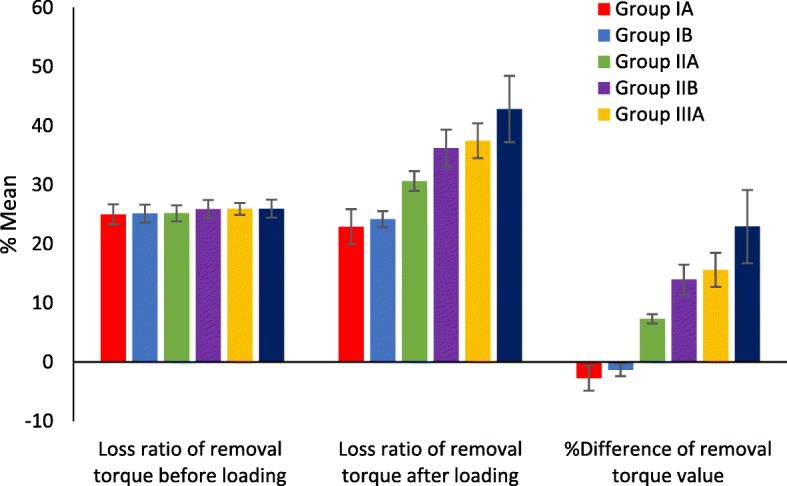
Mean rate ± SD of removal torque loss (%) between groups and results of ANOVA test for loss ratio of removal torque value between groups

**Fig. 6:**
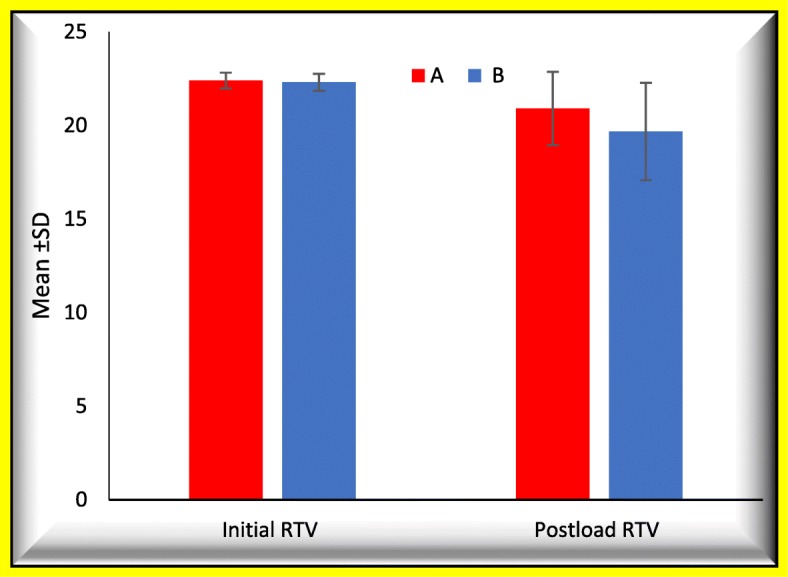
Comparison between short and high collar length (A and B)

One-way ANOVA and pair-wise Tukey’s post hoc tests showed that %initial RTL among the groups was not significantly different (*P* > .05), while %postload RTL was significantly different among the three groups (*P* < .05). GIIIB had the highest %postload RTL (42.8% ± 5.6) while GIA had the lowest (22.8% ± 2.9). For GIA and B, %postload RTL was lower than %initial RTL. %postload RTL is significantly different between different abutment groups (< 0.001*) except between Group IA and Group IB which was not significantly different (*P* > .05). %difference RTL between the three groups is significantly different (< 0.001*) (Tables 1, 2, 3, and 4 and Figs. 4, 5, and 6).

**Table 1:** One-way ANOVA and post hoc Tukey test results for mean ± SD of the %initial RTL, %postload RTL, and %difference between initial and postload RTL between all groups

	%initial RTL	%postload RTL		%difference between initial and postload RTL	
	Mean ± SD				
Subgroups					
GIA	24.9% ± 1.7	22.8% ± 2.9		− 2.7% ± 2.0	
GIB	25.1% ± 1.5	24.1% ± 1.3		− 1.3% ± 1.0	
GIIA	25.1% ± 1.3	30.6% ± 1.6		7.3% ± 0.7	
GIIB	25.9% ± 1.5	36.2% ± 3.1		13.9% ± 2.5	
GIIIA	25.9% ± 1.0	37.4% ± 2.9		15.6% ± 2.8	
GIIIB	25.9% ± 1.5	42.8% ± 5.6		22.9% ± 6.2	
ANOVA					
*F*	1.5	88.0		153.9	
*P* value	0.1	< 0.001*		< 0.001*	
After value	Tukey’s test				
	GIA	GIB	GIIA	GIIB	GIIIA
GIB	0.8				
GIIA	< 0.001*	< 0.001*			
GIIB	< 0.001*	< 0.001*	< 0.001*		
GIIIA	< 0.001*	< 0.001*	< 0.001*	0.9	
GIIIB	< 0.001*	< 0.001*	< 0.001*	< 0.001*	< 0.001*

*−* negative means decreased post loading RTL ratio (removal torque gain)

*statistical significant differences (*P* <.05)

**Table 2:** One-way ANOVA and post hoc Tukey test results for mean ± SD of the initial RTV, postload RTV, and difference between initial and postload RTV between all groups

	Initial RTV	Postload RTV		Difference between initial and postload RTV	
	Mean ± SD				
Subgroups					
GIA	22.5 ± 0.5	23.1 ± 0.8		− 0.6 ± 0.4	
GIB	22.4 ± 0.4	22.7 ± 0.4		− 0.2 ± 0.2	
GIIA	22.4 ± 0.4	20.8 ± 0.5		1.6 ± 0.1	
GIIB	22.2 ± 0.4	19.1 ± 0.9		3.0 ± 0.5	
GIIIA	22.2 ± 0.3	18.7 ± 0.8		3.4 ± 0.5	
GIIIB	22.2 ± 0.4	17.1 ± 1.6		5.0 ± 1.2	
ANOVA					
*F*	1.5	88.0		153.9	
*P* value	0.1	< 0.001*		< 0.001*	
After value	Tukey’s test				
	GIA	GIB	GIIA	GIIB	GIIIA
GIB	0.8				
GIIA	< 0.001*	< 0.001*			
GIIB	< 0.001*	< 0.001*	< 0.001*		
GIIIA	< 0.001*	< 0.001*	< 0.001*	0.9	
GIIIB	< 0.001*	< 0.001*	< 0.001*	< 0.001*	< 0.001*

− negative means decreased post loading RTL ratio (removal torque gain)

*statistical significant differences (*P* <.05)

**Table 3:** Comparison between short and high collar length (A and B)

Collar length		Subgroups		*T* test	
		A (2 mm collar length)	B (4 mm collar length)	*t*	*P* value
Initial RTV	Mean ± SD	22.3 ± 0.4	22.3 ± 0.5	0.9	0.3
Postload RTV	Mean ± SD	20.9 ± 1.9	19.6 ± 2.5	2.5	0.014*
Difference between initial and postload RTV	Mean ± SD	1.4 ± 1.7	2.6 ± 2.3		
Paired test	*P* value	< 0.001*	< 0.001*		

*statistical significant differences (*P* <.05)

**Table 4:** The raw data in all six experimental groups

*N*	Groups	Subgroups	Initial RTV	Postload RTV	Initial RTL	Postload RTL	% of change RTV	% of change RTL
1	GI	A	23	24.1	23.33	19.67	− 4.78	15.69
2	GI	A	22.4	23.04	25.33	23.2	− 2.86	8.41
3	GI	A	22.3	22.9	25.67	23.67	− 2.69	7.79
4	GI	A	21.8	21.5	27.33	28.33	1.38	− 3.66
5	GI	A	23	24.2	23.33	19.33	− 5.22	17.15
6	GI	A	22.1	22.8	26.33	24	− 3.17	8.85
7	GI	A	22.8	23.15	24	22.83	− 1.54	4.88
8	GI	A	22.9	24	23.67	20	− 4.8	15.5
9	GI	A	22.2	22.9	26	23.67	− 3.15	8.96
#	GI	A	22	21.6	26.67	28	1.82	− 4.99
#	GI	A	21.9	22.4	27	25.33	− 2.28	6.19
#	GI	A	22	22.8	26.67	24	− 3.64	10.01
#	GI	A	22.8	23.2	24	22.67	− 1.75	5.54
#	GI	A	22.9	23.9	23.67	20.33	− 4.37	14.11
#	GI	A	23.5	24.5	21.67	18.33	− 4.26	15.41
#	GI	B	23	23.1	23.33	23	− 0.43	1.41
#	GI	B	22.9	23	23.67	23.33	− 0.44	1.44
#	GI	B	22.5	22.7	25	24.33	− 0.89	2.68
#	GI	B	22.4	22.5	25.33	25	− 0.45	1.3
#	GI	B	21.8	22.4	27.33	25.33	− 2.75	7.32
#	GI	B	23	23.1	23.33	23	− 0.43	1.41
#	GI	B	22.8	23	24	23.33	− 0.88	2.79
#	GI	B	22.5	22.7	25	24.33	− 0.89	2.68
#	GI	B	22.7	22.9	24.33	23.67	− 0.88	2.71
#	GI	B	22.8	23.2	24	22.67	− 1.75	5.54
#	GI	B	21.4	21.5	28.67	28.33	− 0.47	1.19
#	GI	B	22.1	22.8	26.33	24	− 3.17	8.85
#	GI	B	22.2	22.97	26	23.43	− 3.47	9.88
#	GI	B	22.5	22.6	25	24.67	− 0.44	1.32
#	GI	B	22.3	22.8	25.67	24	− 2.24	6.51
#	GII	A	22.9	21.4	23.67	28.67	6.55	− 21.12
#	GII	A	22.5	20.9	25	30.33	7.11	− 21.32
#	GII	A	22.4	20.8	25.33	30.67	7.14	− 21.08
#	GII	A	22.1	20.6	26.33	31.33	6.79	− 18.99
#	GII	A	21.9	19.9	27	33.67	9.13	− 24.7
#	GII	A	23	21.5	23.33	28.33	6.52	− 21.43
#	GII	A	22.6	21	24.67	30	7.08	− 21.61
#	GII	A	22.4	20.8	25.33	30.67	7.14	− 21.08
#	GII	A	22	20.5	26.67	31.67	6.82	− 18.75
#	GII	A	22.3	20.7	25.67	31	7.17	− 20.76
#	GII	A	21.8	19.8	27.33	34	9.17	− 24.41
#	GII	A	22.8	21.1	24	29.67	7.46	− 23.63
#	GII	A	22.7	21	24.33	30	7.49	− 23.3
#	GII	A	22.2	20.6	26	31.33	7.21	− 20.5
#	GII	A	23.1	21.5	23	28.33	6.93	− 23.17
#	GII	B	22.8	20.4	24	32	10.53	− 33.33
#	GII	B	22.1	19.2	26.33	36	13.12	− 36.73
#	GII	B	22	18.7	26.67	37.67	15	− 41.24
#	GII	B	21.8	17.8	27.33	40.67	18.35	− 48.81
#	GII	B	22.7	20.1	24.33	33	11.45	− 35.64
#	GII	B	22.1	18.9	26.33	37	14.48	− 40.52
#	GII	B	22.9	20.4	23.67	32	10.92	− 35.19
#	GII	B	22.2	19.4	26	35.33	12.61	− 35.88
#	GII	B	21.5	17.7	28.33	41	17.67	− 44.72
#	GII	B	21.6	17.8	28	40.67	17.59	− 45.25
#	GII	B	22.7	19.5	24.33	35	14.1	− 43.86
#	GII	B	21.9	18.5	27	38.33	15.53	− 41.96
#	GII	B	22.8	20.3	24	32.33	10.96	− 34.71
#	GII	B	22.3	19.5	25.67	35	12.56	− 36.35
#	GII	B	22	18.8	26.67	37.33	14.55	− 39.97
#	GIII	A	22.6	20.1	24.67	33	11.06	− 33.77
#	GIII	A	22.4	19.2	25.33	36	14.29	− 42.12
#	GIII	A	22.3	19.1	25.67	36.33	14.35	− 41.53
#	GIII	A	21.9	17.7	27	41	19.18	− 51.85
#	GIII	A	21.9	17.9	27	40.33	18.26	− 49.37
#	GIII	A	22.5	19.3	25	35.67	14.22	− 42.68
#	GIII	A	22.3	19	25.67	36.67	14.8	− 42.85
#	GIII	A	22.7	20.2	24.33	32.67	11.01	− 34.28
#	GIII	A	21.8	17.8	27.33	40.67	18.35	− 48.81
#	GIII	A	21.8	17.8	27.33	40.67	18.35	− 48.81
#	GIII	A	22	18.1	26.67	39.67	17.73	− 48.74
#	GIII	A	22.2	18.6	26	38	16.22	− 46.15
#	GIII	A	22.6	20.1	24.67	33	11.06	− 33.77
#	GIII	A	22.2	18.3	26	39	17.57	− 50
#	GIII	A	22.1	18.2	26.33	39.33	17.65	− 49.37
#	GIII	B	22.7	18.8	24.33	37.33	17.18	− 53.43
#	GIII	B	22	17	26.67	43.33	22.73	− 62.47
#	GIII	B	22	16.9	26.67	43.67	23.18	− 63.74
#	GIII	B	21.9	15.7	27	47.67	28.31	− 76.56
#	GIII	B	21.8	15.8	27.33	47.33	27.52	− 73.18
#	GIII	B	22.6	18.7	24.67	37.67	17.26	− 52.7
#	GIII	B	22.1	18.2	26.33	39.33	17.65	− 49.37
#	GIII	B	21.7	14.05	27.67	53.17	35.25	− 92.16
#	GIII	B	22	15.9	26.67	47	27.73	− 76.23
#	GIII	B	22.8	18.9	24	37	17.11	− 54.17
#	GIII	B	22.9	19	23.67	36.67	17.03	− 54.92
#	GIII	B	21.6	14.5	28	51.67	32.87	− 84.54
#	GIII	B	23	19.24	23.33	35.87	16.35	− 53.75
#	GIII	B	22	16.8	26.67	44	23.64	− 64.98
#	GIII	B	22.1	17.7	26.33	41	19.91	− 55.72

## Discussion

Conical hybrid connection was used in this study as it was proven that the conical hybrid demonstrated the best stress distribution [20]. This connection has a conical union between the implant fixture and the abutment. In the conical abutment, lateral force is resisted by the taper design of the Morse taper connection. Thus, the stress concentration is resisted by the side-wall contact surface of its taper design. This stress concentration increases at the apical end of the side-wall contact surface where the implant fixture is thicker. This thickness might provide more resistance to the force, especially the off-axis loading force (as in case of angled abutment). Thus, in abutments with conical hybrid connection design, the screw is not the only source of resistance to loading force, as it is in abutments with an internal hexagonal design [21, 22].

In this study, abutment screws were tightened to 30 Ncm according to the manufacturer’s instructions with digital torque gauge. Application of the optimum torque to the implant–abutment complex is critical for long-term successful prosthetic implant restoration. Applied torque develops a force within the screw called preload [23].

Ten-minute interval was left after the first torque application, and all screws were retightened to the same tightening torque (30 Ncm) with the same digital torque gauge to compensate for the preload loss due to settling effect of the screw thus ensure achieving optimal preload as only 10% of the initial torque is transformed into preload, where the remaining 90% is used to overcome the friction between the surface irregularities [23].

The results of this study indicated that there is some torque loss after applying two insertion torques with a 10-min interval before any loading as RTVs were less than 30 Ncm. This finding matches previous studies that reported initial torque loss after 2–10 min [4, 9, 10]. Although there was an increase in %RTL before loading in every group, loosening of screws could not be detected clinically. This may indicate that the remaining tightening torque would serve clinically for a longer period.

In the current study, the results showed that there is a significant difference in %RTL before and after application of dynamic cyclic loading for all angulations and collar lengths. These results are in an agreement with previous researches that found a significant difference between %initial and %postload RTL after mechanical cyclic loading [24, 25]. This result was explained by Bickford et al. [26] as the process of screw loosening occurs in two stages. Initially, external forces cause sliding between the threads, partially relieving the stretching of the screw and reducing preload. At this stage, the higher the preload (within a certain limit), the greater will be the resistance to loosening. The second stage is attained by a gradual reduction of preload below a critical level, in which external forces cause the turning of the screw in an anti-clockwise direction, and it loses its function.

The results of this study showed that, the removal torque loss ratios, with 15° and 25° angulations, were significantly increased. The removal torque loss ratio was increased significantly with increasing angulation as with the 25° angulation the removal torque loss ratio was significantly higher than 15° angulation.

On the other hand, these results disagree with studies that yielded no significant difference between straight and angled abutments for deflection, rotation, and torque required to loosen abutment screws for any parameter at any time [12, 27]. Hsu et al. [28] showed that the clinical performance of angled abutments is comparable to that of straight abutments with respect to both soft tissue responses and general survival rates. However, in vitro studies of stress/strain analyses of angled abutments can only agree that stress/strain levels increase as abutment angulation increases.

These results are in agreement with previous studies which stated that the difference in abutment screw RTV after load showed better results when less angulation abutment was used and studies have failed to show any contraindication to their use [4, 19, 29]. Ha et al. [30], in an evaluation of the influence of abutment angulation on screw loosening of implants in the anterior maxilla, found that the angled abutments showed higher RTV (*P* < .05) than the straight and gold premachined UCLA-type abutments and the difference between them was not significant. This can be attributed to the off-axis force as loading on angled abutments is mostly off-axis, which raises the concern of how angled abutments generally perform with such an unfavorable loading regimen [28].

The increase in RTL with increasing abutment angulation can be attributed to the off-axis force as loading on angled abutments is mostly off-axis, which raises the concern of how angled abutments generally perform with such an unfavorable loading regimen [28]. Forces applied off-axis may be expected to overload the bone surrounding single-tooth implants, as shown by means of finite element analysis, which affects abutment screw leading to its loosening [31].

The greater the angulation, the greater the off-axis force that generates more stress and strain in implant components specially the screw [30] When off-axis loading is applied to an implant, the magnitude of the stress will be increased three times or more [28]. There was a statistically significant increase in stress and strain when abutment angulation increased. This supports the concept of eliminating unnecessary occlusal and off-axial forces on implant-supported restorations [4]. With clinical loading of implants restored using angled abutments, lateral occlusal forces may increase creating torsional force which increases screw loosening [12, 29]. Any direction of load that is not in the long axis of the implant will magnify the crestal stresses to the implant–bone interface and to the abutment screws in the restoration [8].

On the other hand, concerning the area of contact between screw thread and abutment, the increase of abutment angulation leads to decrease area of friction that leads to retention and thus screw loosening occurs. Comparing micromotion level between a straight abutment, a 15° to 25° abutment angulation, an increase in the micromotion level by 30% was observed. This micromotion may explain the screw failure. However, no screw failure occurred in a study with 2261 angled abutment evaluated for 96 months [29, 32].

According to the results of this study, it was showed that with straight abutments, %postload RTL was lower than %initial RTL. This result could be explained by Squier et al. [33] who stated that abutments of the conical hybrid connection showed detorque values higher than the initial torque due to the cold solder on the implant–abutment interface, which agrees with the results of this study. This condition arises from the friction between the two surfaces, which differ slightly; the pressure created by the insertion force determines the maintenance of the connection even after stopping the applied force for insertion.

Several studies have been conducted on this type of implant–abutment connection [23, 34]. Sutter et al. [35] demonstrated reverse torque values of this hybrid implant–abutment connection that were 124% of the initial tightening torque. These authors suggested that cold welding occurred in the conical hybrid implant–abutment connection.

Schmitt et al. [36] compared conical and nonconical implant–abutment connection systems in terms of their in vitro and in vivo performances. In vitro studies indicate that conical and nonconical abutments exhibited sufficient resistance to the maximal bending forces and fatigue loading. However, conical abutments were superior in terms of sealing, microgap formation, torque maintenance, and abutment stability.

According to the results of this study, %RTL increased significantly with increasing collar length and the percentage of removal torque gaining after load with 0° abutments with collar height 4 mm was less than that of 2 mm. This finding matches what was found by Siadat et al. [9] who evaluated the effect of collar length on screw loosening and concluded that an increase in the abutment collar length significantly increase the torque loss of abutment–implant screw after cyclic loading. The abutment collar length acts as vertical cantilever, so increasing abutment collar would lead to an increased vertical cantilever which acts as a force magnifier [8]. Cantilever designs increase bending forces on screws due to the lever effect [17, 18].

## Conclusions

Within the limitations of this in vitro study, it can be concluded that:

- Screw loosening increases with increasing abutment angulation and collar length after 100,000 cycles of dynamic cyclic loading.
- Results of this study showed that conical hybrid connection design provides more biomechanically stable screw joint with straight abutments than angled abutments.

## References

[1] Prado CJ, Neves FD, Soares CJ, Dantas KA, Dantas TS, Naves LZ (2014). Influence of abutment screw design and surface coating on the bending flexural strength of the implant set. J Oral Implantol..

[2] Goodacre CJ, Bernal G, Rungcharassaeng K, Kan JY (2003). Clinical complications with implants and implant prostheses. J Prosthet Dent..

[3] Michalakis KX, Calvani PL, Muftu S, Pissiotis A, Hirayama H (2014). The effect of different implant-abutment connections on screw joint stability. J Oral Implantol..

[4] Asvanund P, Cheepsathit L (2016). Effect of different angulation angled abutment on screw loosening of implants under cyclic loading. M Dent J..

[5] Ferreira MB, Delben JA, Barao VA, Faverani LP, Dos Santos PH, Assuncao WG (2012). Evaluation of torque maintenance of abutment and cylinder screws with Morse taper implants. J Craniofac Surg..

[6] Geckili E, Geckili O, Bilhan H, Kutay O, Bilgin T (2017). Clinical comparison of screw-retained and screwless Morse taper implant-abutment connections: one-year postloading results. Int J Oral Maxillofac Implants..

[7] Schwarz MS (2000). Mechanical complications of dental implants. Clin Oral Implants Res..

[8] Misch CE (2015). Principles for abutment and prosthetic screws and screw-retained components and prostheses. Elsevier Mosby.

[9] Siadat H, Pirmoazen S, Beyabanaki E, Alikhasi M (2015). Does abutment collar length affect abutment screw loosening after cyclic loading?. J Oral Implantol..

[10] Delben JA, Gomes EA, Barao VA, Assuncao WG (2011). Evaluation of the effect of retightening and mechanical cycling on preload maintenance of retention screws. Int J Oral Maxillofac Implants..

[11] Cho WR, Huh YH, Park CJ, Cho LR (2015). Effect of cyclic loading and retightening on reverse torque value in external and internal implants. J Adv Prosthodont..

[12] Eger DE, Gunsolley JC, Feldman S (2000). Comparison of angled and standard abutments and their effect on clinical outcomes: a preliminary report. Int J Oral Maxillofac Implants..

[13] Antoun H, Belmon P, Cherfane P, Sitbon JM (2012). Immediate loading of four or six implants in completely edentulous patients. Int J Periodontics Restorative Dent..

[14] Cavallaro JG, Greenstein G (2011). Angled implant abutments. A Practical Application of Available Knowledge J Am Dent Assoc..

[15] Kallus T, Henry P, Jemt T, Jorneus L (1990). Clinical evaluation of angulated abutments for the Branemark system: a pilot study. Int J Oral Maxillofac Implants..

[16] Herrero-Climent M, Romero-Ruiz MM, Diaz-Castro CM, Bullon P, Rios-Santos JV (2014). Influence of two different machined-collar heights on crestal bone loss. Int J Oral Maxillofac Implants..

[17] Stuker RA, Teixeira ER, Beck JP, Costa NP (2008). Preload and torque removal evaluation of three different abutment screws for single standing implant restorations. J Appl Oral Sci..

[18] Kim NG, Kim YS, Kim CW, Jang KS, Lim YJ (2004). The effect of abutment height on screw loosening in single implant-supported prothesis after dynamic cyclic loading. J Korean Acad Prosthodont..

[19] Sethi A, Kaus T, Sochor P (2000). The use of angulated abutments in implant dentistry: five-year clinical results of an ongoing prospective study. Int J Oral Maxillofac Implants..

[20] Tonella BP, Pellizzer EP, Ferraco R, Falcon-Antenucci RM, Carvalho PS, Goiato MC (2011). Photoelastic analysis of cemented or screwed implant-supported prostheses with different prosthetic connections. J Oral Implantol..

[21] Akca K, Cehreli MC, Iplikcioglu H (2003). Evaluation of the mechanical characteristics of the implant abutment complex of a reduced-diameter Morsetaper implant. A nonlinear finite element stress analysis. Clin Oral Implants Res..

[22] Bozkaya D, Muftu S (2003). Mechanics of the tapered interference fit in dental implants. J Biomech..

[23] Pardal-Pelaez B, Montero J (2017). Preload loss of abutment screws after dynamic fatigue in single implant-supported restorations. A systematic review. J Clin Exp Dent..

[24] Mohammed HH, Lee JH, Bae JM, Cho HW (2016). Effect of abutment screw length and cyclic loading on removal torque in external and internal hex implants. J Adv Prosthodont..

[25] Shin HM, Huh JB, Yun MJ, Jeon YC, Chang BM, Jeong CM (2014). Influence of the implant-abutment connection design and diameter on the screw joint stability. J Adv Prosthodont..

[26] Bickford JH, Nassar S (1998). Handbook of bolts and bolted joints.

[27] Dixon DL, Breeding LC, Sadler JP, McKay ML (1995). Comparison of screw loosening, rotation, and deflection among three implant designs. J Prosthet Dent..

[28] Hsu ML, Chung TF, Kao HC (2005). Clinical applications of angled abutments - a literature review. Chin Dent J..

[29] Morsch CS, Rafael CF, Dumes HF, Juanito GM, Bianchini MA (2015). Failure of prosthetic screws on 971 implants. Braz J Oral Sci..

[30] Ha CY, Lim YJ, Kim MJ, Choi JH (2011). The influence of abutment angulation on screw loosening of implants in the anterior maxilla. Int J Oral Maxillofac Implants..

[31] Papavasiliou G, Kamposiora P, Bayne SC, Felton DA (1996). Three-dimensional finite element analysis of stress-distribution around single tooth implants as a function of bony support, prosthesis type, and loading during function. J Prosthet Dent..

[32] Szmukler-Moncler S, Salama H, Reingewirtz Y, Dubruille JH (1998). Timing of loading and effect of micromotion on bone-dental implant interface: review of experimental literature. J BiomedMater Res..

[33] Squier RS, Psoter WJ, Taylor TD (2002). Removal torques of conical, tapered implant abutments: the effects of anodization and reduction of surface area. Int J Oral Maxillofac Implants..

[34] Norton MR (2000). In vitro evaluation of the strength of the conical implant to abutment joint in two commercially available implant systems. J Preosthet Dent..

[35] Sutter F, Weber HP, Sorensen J, Belser U (1993). The new restorative concept of the ITI dental implant system: design and engineering. Int J Periodontics Restorative Dent..

[36] Schmitt CM, Nogueira-Filho G, Tenenbaum HC, Lai JY, Brito C, Doring H (2014). Performance of conical abutment (Morse taper) connection implants: a systematic review. J Biomed Mater Res A..

